# Sexually transmitted infection screening, prevalence and incidence among South African men and transgender women who have sex with men enrolled in a combination HIV prevention cohort study: the Sibanye Methods for Prevention Packages Programme (MP3) project

**DOI:** 10.1002/jia2.25594

**Published:** 2020-10-01

**Authors:** Jeb Jones, Travis H Sanchez, Karen Dominguez, Linda‐Gail Bekker, Nancy Phaswana‐Mafuya, Stefan D Baral, AD McNaghten, Lesego B Kgatitswe, Rachel Valencia, Clarence S Yah, Ryan Zahn, Aaron J Siegler, Patrick S Sullivan

**Affiliations:** ^1^ Emory University Atlanta GA USA; ^2^ Desmond Tutu HIV Centre University of Cape Town Observatory South Africa; ^3^ North West University Potchefstroom South Africa; ^4^ Human Sciences Research Council of South Africa Pretoria South Africa; ^5^ Johns Hopkins University School of Public Health Baltimore MD USA; ^6^ Wits Reproductive Health and HIV Institute Faculty of Health Sciences University of the Witwatersrand Johannesburg South Africa; ^7^ School of Heath Systems and Public Health University of Pretoria Pretoria South Africa

**Keywords:** men who have sex with men, chlamydia, gonorrhoea, syphilis, STI testing, STI incidence, HIV, MSM/TGW, sexually transmitted infections, chlamydia, gonorrhoea, syphilis

## Abstract

**Introduction:**

Men who have sex with men (MSM) and transgender women (TGW) experience high incidence and prevalence of sexually transmitted infections (STI), and data are needed to understand risk factors for STIs in these populations. The Sibanye Health Project was conducted in Cape Town and Port Elizabeth, South Africa from 2015 to 2016 to develop and test a package of HIV prevention interventions for MSM and TGW. We describe the incidence, prevalence and symptoms of *Chlamydia trachomatis* (CT), *Neisseria gonorrhea* (NG) and syphilis observed during the study.

**Methods:**

Participants completed HIV testing at baseline. All participants who were HIV negative were followed prospectively. Additionally, a sample of participants identified as living with HIV at baseline was selected to be followed prospectively so that the prospective cohort was approximately 20% HIV positive; the remaining participants identified as HIV positive at baseline were not followed prospectively. Prospective participants were followed for 12 months and returned for clinic‐based STI/HIV testing and assessment of STI symptoms at months 6 and 12. Additional HIV/STI testing visits could be scheduled at participant request.

**Results:**

Following consent, a total of 292 participants attended a baseline visit (mean age = 26 years), and 201 were enrolled for the 12‐month prospective study. Acceptance of screening for syphilis and urethral NG/CT was near universal, though acceptance of screening for rectal NG/CT was lower (194/292; 66%). Prevalence of urethral CT and NG at baseline was 10% (29/289) and 3% (8/288) respectively; incidence of urethral CT and NG was 12.8/100 person‐years (PY) and 7.1/100 PY respectively. Prevalence of rectal CT and NG at baseline was 25% (47/189) and 16% (30/189) respectively; incidence of rectal CT and NG was 33.4/100 PY and 26.8/100 PY respectively. Prevalence of syphilis at baseline was 17% (45/258) and incidence was 8.2/100 PY. 91%, 95% and 97% of diagnosed rectal NG/CT, urethral NG/CT and syphilis infections, respectively, were clinically asymptomatic.

**Conclusions:**

Prevalence and incidence of urethral and rectal STIs were high among these South African MSM and TGW, and were similar to rates in other settings in the world. Clinical symptoms from these infections were rare, highlighting limitations of syndromic surveillance and suggesting the need for presumptive testing and/or treatment to address the STI epidemic among MSM/TGW in South Africa.

## 1 INTRODUCTION

There is evidence worldwide that men who have sex with men (MSM) and transgender women (TGW) experience high rates of sexually transmitted infections (STIs), such as syphilis, *Chlamydia trachomatis* (CT) and *Neisseria gonorrhea* (NG) [1, 2]. The South African National Strategic Plan on HIV/AIDS, Tuberculosis and STIs identifies MSM and TGW as key populations [3]. Many STIs – especially rectal STIs in MSM and TGW – are asymptomatic [4], and timely diagnosis and treatment for individual and public health benefits requires routine screening [5]. Current STI screening recommendations in South Africa are based on syndromic surveillance and management [6]. Much of the STI screening that is conducted with MSM and TGW is by blood or urine specimens, but in some studies of MSM and TGW, there is considerably higher prevalence of rectal STIs than urethral STIs or syphilis [7, 8, 9] and multi‐site screening has been shown to substantially increase the yield of positive tests [10].

Despite a well‐described body of research on STI prevalence among MSM [11, 12, 13], there are few published studies of incident STI infection among MSM and TGW that examine factors associated with STI acquisition. These data are needed to draw stronger inferences about risk factors for STI acquisition and potential intervention targets for STI prevention. A recent study in the Netherlands identified partner age, HIV infection and sex following alcohol consumption as risk factors for incident STIs among MSM [14]. The dearth of data may be due to the complexity in differentiating persistent STI infection from true STI incidence, which is likely only feasible in a prospective research design in which treatment can be verified. These studies have not been commonly undertaken solely for examining STI incidence, but have been conducted within HIV prevention or epidemiological research [12, 15].

The Sibanye Health Project, a pilot study of a comprehensive HIV prevention programme for MSM and TGW, was conducted in Cape Town and Port Elizabeth, South Africa. The project enrolled and prospectively followed a cohort of MSM and TGW who selected from a suite of HIV prevention services that included STI screening and treatment [16]. In this study, we examined screening acceptance, STI prevalence and incidence and treatment of diagnosed STIs. We also assessed factors associated with STI prevalence and predictive of incident STI diagnosis.

## 2 METHODS

### 2.1 Study population and procedures

The Sibanye Health Project was conducted in Cape Town and Port Elizabeth, South Africa from 2015 to 2016 to develop a package of HIV prevention interventions for MSM and TGW in South Africa and to conduct a pilot study to test the package of interventions [16]. Eligible participants were at least 18 years old, had anal sex with a man in the previous 12 months, resided in Cape Town or Port Elizabeth with plans to stay in the city for the next year, could complete surveys in English, Xhosa or Afrikaans, were assigned male sex at birth, were willing to provide contact information, and had a phone to facilitate scheduling study visits. Participants who identified as any gender other than male were classified as TGW.

All eligible participants completed a baseline visit and were included in the baseline cohort. All HIV negative and a sample of HIV‐positive participants were then enrolled into a prospective cohort. The prospective cohort was designed to be 20% MSM and TGW currently living with HIV, with the remainder at risk of HIV. The remaining participants who were HIV positive at the baseline visit were not enrolled in the prospective cohort. Prospective participants were followed for one year and completed STI screening at 6‐ and 12‐month timepoints. Participants were compensated R65 for each of these study visits and up to R60 for transport to attend study visits. Additional *ad hoc* visits also occurred for patients who initiated pre‐exposure prophylaxis (PrEP) or who requested STI testing and/or treatment. Tests for syphilis and rectal and urethral CT and NG were conducted at baseline, month 6 and month 12. Participants could choose to opt out of testing.

This study was approved by the Institutional Review Board of Emory University, the University of Cape Town Institutional Review Board and the Research Ethics Committee of the Human Sciences Research Council. Informed consent was obtained from participants at the beginning of the baseline study visit.

### 2.2 Measures

STI testing was conducted at baseline, month 6, and month 12. Syphilis testing was performed using the syphilis rapid plasma reagin (RPR) test. Positive test results were confirmed with titres and *T pallidum* particle agglutination (TPPA). Urine was self‐collected and rectal swabs were taken by clinician direct swabbing to obtain samples for CT and NG testing. CT and NG were diagnosed using the Cepheid GeneXpert NG/CT test in Cape Town and Gen‐Probe Aptima Assay in Port Elizabeth. A clinical exam and patient history were also conducted at all visits to assess the extent to which STIs were symptomatic. Visual genital inspections were conducted to note the presence of urethral or perianal STI signs/symptoms (urethral symptoms: urethral discharge and painful/burning sensation during urination; rectal symptoms: rectal discharge, anal itching and painful bowel movements). Syphilis signs/symptoms included ulcers on the genitals, rectum or buttocks and vesicles in the rectal or groin area. Diagnoses were made based on laboratory results; participants who received an STI diagnosis were provided appropriate treatment or referred to a local clinic.

Demographic and behavioural data were collected via self‐administered surveys at all study visits. Participants reported age, race, gender and sexual identity, highest educational attainment, work/student status, and income. Relevant behavioural variables included sexual risk factors (receptive condomless anal intercourse, number of male and female partners, transactional sex) and substance use.

### 2.3 Analyses

Acceptance of NG/CT screening was defined as agreement for specimen collection for screening by anatomical site. Syphilis testing was conducted routinely as part of the blood collection performed at the scheduled visits; thus, acceptance of syphilis testing was defined as agreement for blood collection. Acceptance of urethral and rectal STI specimen collection for screening is reported at baseline (all enrolled participants), at any point during the twelve‐month follow‐up and at the six‐ and twelve‐month visits specifically (prospectively enrolled participants only). We present uptake at any point during the 12‐month period because screening could occur at *ad hoc* visits outside the 6‐monthly visit schedule.

STIs detected at the baseline study visit were considered to be prevalent infections. If treatment of diagnosed STIs was confirmed, subsequent STIs were considered to be incident infections. Concurrent STIs were identified if a participant was infected with more than one organism at the same time point or infection with the same organism at more than one anatomical site. STI prevalence for urethral and rectal CT, urethral and rectal NG, and syphilis are reported at baseline for all enrolled participants, regardless of whether they were enrolled in the prospective cohort.

Unadjusted associations between STI screening acceptance and prevalence and demographic, clinical and behavioural factors were assessed via chi square tests except when expected cell values were small and Fisher exact tests were used. We used Poisson regression with robust variance [17] to estimate prevalence ratios (PR) comparing acceptance adjusted for study site and other factors found to be statistically significant (*p* < 0.05) in bivariate analyses.

STI incidence for urethral and rectal chlamydia, urethral and rectal gonorrhoea, and syphilis are reported for prospectively enrolled participants. STI incidence rates are expressed as number of incident infections per 100 person‐years (PY) at risk. Person‐years of follow‐up were determined by totalling the number of days of observation for those who were at risk of STI infection for each anatomical site and STI combination. We considered participants at risk for a given combination if they had no evidence of prevalent infection during the follow‐up period (e.g. untreated infection). The time period a participant was on treatment was excluded from the at‐risk period. Rates and rate ratios of incident NG, CT and syphilis infections were modelled using Poisson regression. Because MSM and TGW are heterogenous populations and the study population was predominantly composed of MSM, we present a sensitivity analysis of incidence rates and rate ratios restricted to MSM in Table S2.

Additional descriptive analyses are reported to describe the proportion of laboratory diagnosed urethral and rectal chlamydia, urethral and rectal gonorrhoea, and syphilis infections that were symptomatic, concurrent at the baseline visit, and successfully treated. All analyses were conducted using SAS 9.4 (SAS Institute, Cary, NC, USA). Statistical significance was determined at *p* < 0.05.

## 3 RESULTS

A total of 292 (115 in Cape Town, 177 in Port Elizabeth) participants were enrolled, 201 (100 in Cape Town, 101 in Port Elizabeth) of whom were followed prospectively (Table 1). The prospective participants were composed of all HIV‐negative participants and a sample of HIV‐positive participants such that the HIV prevalence in the prospective cohort was approximately 20% at the beginning of follow‐up. Most participants identified as Black (254/292; 87%), male (263/285; 92%) and gay (192/287; 67%); a total of 22 (7.7%) participants were TGW. The prevalence of HIV was 31% (91/292) at baseline. Overall, 11% of participants had two or more concurrent STIs at baseline. Among prospective participants, 86% (172/201) and 87% (174/201) completed study visits at months 6 and 12 respectively.

**Table 1:** Demographic characteristics of men who have sex with men and transgender women enrolled for baseline (N = 292) and prospective follow‐up (N = 201) in Cape Town and Port Elizabeth, South Africa

	Total	Baseline Only Participants	Prospective Participants
	N	N (%)	N (%)
Site			
Cape Town	115	15 (16.5)	100 (49.8)
Port Elizabeth	177	76 (83.5)	101 (50.3)
Age			
18 to 24	165	43 (47.3)	122 (60.7)
25+	127	48 (52.8)	79 (39.3)
Race			
Black	254	89 (87.0)	165 (82.1)
Other	38	2 (13.0)	36 (17.9)
Gender identity			
Male	263	83 (91.2)	180 (88.6)
Transgender or other non‐male identified	22	6 (8.8)	16 (11.4)
Sexual identity			
Gay/homosexual	192	77 (85.6)	115 (57.9)
Bisexual, heterosexual, or other	95	13 (14.4)	82 (42.1)
Education^a^			
Did not matriculate	137	36 (40.0)	101 (51.0)
Matriculate or higher	151	54 (60.0)	97 (49.0)
Work/student status			
Part/full‐time student or part/full‐time job	150	49 (54.4)	101 (51.3)
Not a student and no job	137	41 (45.6)	96 (48.7)
Income			
No income	141	43 (50.6)	98 (52.7)
Any income	130	42 (49.4)	88 (47.3)
Baseline HIV status^b^			
Negative	167	–	167 (83.1)
Positive	125	91 (100.0)	34 (16.9)
Initiated PrEP During follow‐up			
No	85	–	85 (50.9)
Yes	82	–	82 (49.1)
Receptive condomless anal intercourse, past three months			
No	165	39 (47.0)	126 (72.0)
Yes	93	44 (53.0)	49 (28.0)
Number of male partners, past three months			
0 to 2	212	72 (81.9)	140 (81.9)
3+	47	16 (18.2)	31 (18.1)
Any female partners, past 12 months			
No	230	81 (89.0)	149 (73.4)
Yes	60	10 (11.0)	50 (26.6)
Transactional sex, past 12 months			
No	225	76 (90.5)	149 (76.4)
Yes	43	8 (9.5)	35 (23.6)
Injection drug use, past six months			
No	72	16 (94.1)	56 (90.3)
Yes	7	1 (5.9)	6 (9.7)
Any drug use, past six months			
No	211	74 (81.3)	137 (68.8)
Yes	79	17 (18.7)	62 (31.2)
Binge drinking (5 + drinks) on 5 or more days, past 30 days			
No	215	65 (75.6)	150 (80.7)
Yes	57	21 (24.4)	36 (19.4)

All participants completed a baseline visit. All HIV‐negative participants and a sample of HIV‐positive participants were followed prospectively. PrEP, pre‐exposure prophylaxis.

^a^ Did not matriculate indicates not completing high school; Matriculate or higher indicates high school graduate or above

^b^ Baseline‐only participants were all HIV positive.

### 3.1 Baseline screening and STI prevalence

Of 292 participants enrolled in baseline procedures, there was universal acceptance of urethral (292/292; 100%) screening, near‐universal acceptance of syphilis (289/292; 99%) screening, and 189 (64.7%) accepted rectal STI screening (Table 2). Baseline rectal screening was more likely to be accepted among participants in Cape Town compared to Port Elizabeth (93.9% vs. 48.6%, *p* < 0.01) and those who identified as gay compared to some other sexual identity (71.4% vs. 55.8%, *p* = 0.01). In adjusted models, only age group was significantly associated with acceptance: acceptance of rectal screening was higher among 18‐ to 24‐year‐old participants [PR = 1.1, 95% confidence interval (CI): 1.0, 1.2] compared to participants age 25 and over. Baseline urethral and syphilis screening did not significantly differ by site, participant characteristics or behaviours (Table S1).

**Table 2:** Acceptance of Rectal STI screening at baseline and over 12 months of follow‐up among men who have sex with men and transgender women in Cape Town and Port Elizabeth, South Africa

	Rectal STI screening acceptance							
	Baseline (N = 292)		Follow‐up^a^ (N = 189)		6 Month Visit (N = 172)		12 Month Visit (N = 174)	
	Prevalence (95% CI)	*p*‐value	Prevalence (95% CI)	*p*‐value	Prevalence (95% CI)	*p*‐value	Prevalence (95% CI)	*p*‐value
Site								
Cape Town	93.9 (89.5, 98.3)	<0.01	83.0 (75.4, 90.6)	0.04	82.1 (74.0, 90.3)	0.08	64.4 (54.3, 74.4)	0.12
Port Elizabeth	48.6 (41.2, 56.0)		69.5 (60.2, 78.7)		70.5 (60.9, 80.0)		51.7 (41.2, 62.2)	
Age ranges								
18 to 24	67.3 (60.1, 74.4)	0.73	77.0 (67.4, 86.6)	0.86	76.4 (68.3, 84.5)	1.00	57.0 (47.6, 66.4)	0.75
25+	65.4 (57.1, 73.6)		75.7 (67.8, 83.5)		75.8 (65.4, 86.1)		59.7 (48.0, 71.4)	
Race								
Black	66.1 (60.3, 72.0)	0.78	75.3 (68.5, 82.1)	0.66	77.9 (71.0, 84.7)	0.36	56.7 (48.6, 64.9)	0.56
Other	68.4 (53.6, 83.2)		80.0 (66.7, 93.3)		68.8 (52.7, 84.8)		63.6 (47.2, 80.0)	
Gender identity								
Male	66.9 (61.2, 72.6)	0.75	74.6 (68.0, 81.1)	0.12	75.0 (68.1, 81.9)	0.53	55.2 (47.3, 63.0)	0.10
Other	63.6 (43.5, 83.7)		93.3 (80.7, 100.0)		86.7 (69.5, 100.0)		80.0 (59.8, 100.0)	
Sexual identity								
Gay/homosexual	71.4 (65.0, 77.7)	0.01	85.2 (78.5, 91.9)	<0.01	85.0 (78.0, 92.0)	<0.01	62.0 (52.5, 71.5)	0.16
Bisexual or other	55.8 (45.8, 65.8)		62.8 (52.1, 73.5)		62.3 (50.9, 73.8)		50.7 (39.1, 62.3)	
Education^b^								
Did not matriculate	63.5 (55.4, 71.6)	0.40	80.6 (72.6, 88.7)	0.17	80.2 (71.8, 88.6)	0.21	56.8 (46.5, 67.2)	1.00
Matriculate or higher	68.2 (60.8, 75.6)		71.0 (61.7, 80.2)		71.1 (61.3, 80.8)		57.8 (47.2, 68.5)	
Combined work/student								
Part/full‐time student or part/full‐time job	71.3 (64.1, 78.6)	0.07	80.2 (72.2, 88.2)	0.23	80.5 (72.1, 88.8)	0.21	63.6 (53.6, 73.7)	0.12
Not a student and no job	61.3 (53.2, 69.5)		71.9 (62.6, 81.2)		71.6 (61.8, 81.4)		51.2 (40.4, 62.0)	
Income								
No income	61.0 (52.9, 69.0)	0.02	75.6 (66.7, 84.4)	0.86	77.5 (68.3, 86.7)	0.85	61.7 (51.1, 72.3)	0.42
Any income	73.8 (66.3, 81.4)		77.6 (68.8, 86.5)		75.6 (66.1, 85.2)		54.4 (43.4, 65.4)	
Baseline HIV status								
Negative	66.5 (59.3, 73.6)	0.99	75.6 (68.9, 82.4)	0.82	75.5 (68.5, 82.6)	0.81	56.6 (48.5, 64.8)	0.55
Positive	66.4 (58.1, 74.7)		78.8 (64.8, 92.7)		79.3 (64.6, 94.1)		64.5 (47.7, 81.4)	
Initiated PrEP during follow‐up								
No	60.0 (49.6, 70.4)	0.07	73.7 (63.8, 83.6)	0.71	78.8 (68.9, 88.7)	0.44	58.2 (46.4, 70.0)	0.74
Yes	73.2 (63.6, 82.8)		77.5 (68.3, 86.7)		72.7 (62.8, 82.7)		55.3 (44.1, 66.4)	
Receptive condomless anal intercourse, past three months								
No	64.2 (56.9, 71.6)	0.27	74.1 (66.2, 82.1)	0.42	74.0 (65.6, 82.5)	0.28	57.5 (48.1, 67.0)	0.36
Yes	71.0 (61.7, 80.2)		81.3 (70.2, 92.3)		83.7 (72.7, 94.8)		66.7 (52.9, 80.4)	
Number of male partners in past three months								
0 to 2	64.6 (58.2, 71.1)	0.11	76.7 (69.5, 83.9)	0.62	75.8 (68.3, 83.3)	0.60	56.5 (47.7, 65.2)	0.28
3+	76.6 (64.5, 88.7)		82.1 (68.0, 96.3)		83.3 (68.4, 98.2)		69.2 (51.5, 87.0)	
Any female partners, past 12 months								
No	68.7 (62.7, 74.7)	0.08	82.0 (75.6, 88.4)	<0.01	80.6 (73.8, 87.4)	0.02	64.1 (55.9, 72.3)	0.01
Yes	56.7 (44.1, 69.2)		58.3 (44.4, 72.3)		61.0 (46.0, 75.9)		39.0 (24.1, 54.0)	
Transactional sex, past 12 months								
No	64.0 (57.7, 70.3)	0.19	76.1 (69.0, 83.2)	1.00	75.4 (67.9, 82.9)	1.00	59.7 (51.2, 68.2)	0.30
Yes	74.4 (61.4, 87.5)		76.5 (62.2, 90.7)		76.7 (61.5, 91.8)		48.3 (30.1, 66.5)	
Injection drug use, past six months								
No	59.7 (48.4, 71.1)	1.00	72.7 (61.0, 84.5)	0.32	72.0 (59.6, 84.4)	0.31	52.9 (39.2, 66.6)	0.68
Yes	57.1 (20.5, 93.8)		100.0 (100.0, 100.0)		100.0 (100.0, 100.0)		66.7 (28.9, 100.0)	
Any drug use, past six months								
No	69.2 (63.0, 75.4)	0.12	76.2 (68.8, 83.6)	1.00	76.5 (68.8, 84.3)	0.85	59.1 (50.1, 68.1)	0.62
Yes	59.5 (48.7, 70.3)		75.4 (64.6, 86.2)		74.5 (63.0, 86.1)		54.4 (41.5, 67.3)	
Binge drinking (5 + drinks) on 5 or more days, past 30 days								
No	66.0 (59.7, 72.4)	0.74	76.4 (69.4, 83.5)	0.83	76.0 (68.6, 83.3)	1.00	54.5 (46.1, 63.0)	0.23
Yes	68.4 (56.4, 80.5)		74.3 (59.8, 88.8)		77.4 (62.7, 92.1)		67.7 (51.3, 84.2)	

All participants are included in the baseline estimates; only prospective participants are included in the follow‐up estimates. Prospective participants are all HIV‐negative participants and a sample of HIV‐positive participants. CI, confidence interval; PrEP, pre‐exposure prophylaxis; STI, sexually transmitted infections.

^a^ Follow‐up prevalence column indicates any uptake over 12 months of follow‐up, columns for month 6 and month 12 indicate uptake at those visits specifically

^b^ Did not matriculate indicates not completing high school; Matriculate or higher indicates high school graduate or above.

Among 289 participants screened for urethral STI at baseline, 29 (10%) had urethral CT infection and 8 (3%) had urethral NG infection (Table 3). Among MSM, the prevalence of urethral CT was 10.8% (95% CI: 7.0, 14.5) and urethral GC was 2.3% (95% CI: 0.5, 4.1). Among TGW, the prevalence of urethral CT was 4.5% (95% CI: 0.0, 13.2) and urethral GC was 4.8% (95% CI: 0.0, 13.9). Prevalent urethral CT infection was associated in crude analyses with baseline HIV status and receptive condomless anal intercourse in the past three months; prevalent urethral NG infection was associated in crude analyses with sexual identity and having any female sex partners in the past 12 months. None of the observed associations with prevalent urethral NG or CT remained statistically significant in adjusted models.

**Table 3:** Prevalence and 95% confidence intervals of urethral and rectal chlamydia, urethral and rectal gonorrhoea, and syphilis among 292 men who have sex with men and transgender women in Cape Town and Port Elizabeth, South Africa

	Chlamydia				Gonorrhoea				Syphilis	
	Rectal (N = 189)		Urethral (N = 270)		Rectal (N = 189)		Urethral (N = 288)		(N = 288)	
	Prevalence (95% CI)	*p*‐value	Prevalence (95% CI)	*p*‐value	Prevalence (95% CI)	*p*‐value	Prevalence (95% CI)	*p*‐value	Prevalence (95% CI)	*p*‐value
Site										
Cape Town	26.4 (18.0, 34.8)	0.61	9.6 (4.2, 15.1)	1.00	18.9 (11.4, 26.3)	0.23	5.3 (1.2, 9.4)	0.06	17.4 (10.5, 24.3)	0.19
Port Elizabeth	22.9 (13.9, 31.9)		10.3 (5.8, 14.8)		12.0 (5.0, 19.1)		1.1 (0.0, 2.7)		24.3 (17.9, 30.7)	
Age										
18 to 24	34.3 (25.3, 43.2)	<0.01	7.9 (3.8, 12.0)	0.17	22.2 (14.4, 30.1)	0.01	3.6 (0.8, 6.5)	0.47	15.3 (9.8, 20.9)	<0.01
25+	12.3 (5.2, 19.5)		12.9 (7.0, 18.8)		7.4 (1.7, 13.1)		1.6 (0.0, 3.9)		29.6 (21.6, 37.6)	
Race										
Black	26.7 (19.9, 33.4)	0.20	9.9 (6.2, 13.6)	0.77	15.8 (10.2, 21.3)	1.00	2.8 (0.8, 4.8)	1.00	22.0 (16.9, 27.1)	0.83
Other	12.5 (0.0, 25.7)		10.8 (0.8, 20.8)		16.7 (1.8, 31.6)		2.7 (0.0, 7.9)		18.4 (6.1, 30.7)	
Gender identity										
Male	24.0 (17.6, 30.4)	0.75	10.8 (7.0, 14.5)	0.71	15.2 (9.8, 20.6)	0.46	2.3 (0.5, 4.1)	0.42	21.6 (16.6, 26.6)	1.00
Transgender or other non‐male identified	28.6 (4.9, 52.2)		4.5 (0.0, 13.2)		21.4 (0.0, 42.9)		4.8 (0.0, 13.9)		18.2 (2.1, 34.3)	
Sexual identity										
Gay/homosexual	29.3 (21.6, 37.1)	0.02	7.9 (4.1, 11.7)	0.09	18.0 (11.5, 24.6)	0.18	1.1 (0.0, 2.5)	0.04	25.4 (19.2, 31.6)	0.03
Bisexual, heterosexual, or other	13.5 (4.2, 22.7)		14.9 (7.7, 22.1)		9.6 (1.6, 17.6)		5.3 (0.8, 9.9)		13.7 (6.8, 20.6)	
Education^a^										
Did notmatriculate	20.9 (12.3, 29.5)	0.39	9.6 (4.7, 14.6)	0.85	14.0 (6.6, 21.3)	0.84	1.5 (0.0, 3.5)	0.45	22.1 (15.1, 29.0)	0.89
Matriculate or Higher	27.3 (18.5, 36.0)		10.7 (5.7, 15.6)		16.2 (8.9, 23.4)		3.3 (0.5, 6.2)		20.9 (14.4, 27.5)	
Work/student status										
Part/full‐time student or part/full‐time job	27.5 (18.8, 36.1)	0.40	8.8 (4.2, 13.3)	0.44	12.7 (6.3, 19.2)	0.31	2.7 (0.1, 5.4)	1.00	20.1 (13.7, 26.6)	0.57
Not a student and no job	21.4 (12.7, 30.2)		11.8 (6.3, 17.2)		19.0 (10.7, 27.4)		2.2 (0.0, 4.7)		23.1 (16.0, 30.3)	
Income										
No income	22.1 (13.3, 30.9)	0.86	10.9 (5.5, 16.2)	0.84	19.8 (11.4, 28.2)	0.14	2.2 (0.0, 4.6)	0.71	24.5 (17.3, 31.6)	0.38
Any income	24.2 (15.4, 33.0)		10.1 (5.1, 15.1)		11.0 (4.6, 17.4)		3.1 (0.1, 6.1)		19.5 (12.7, 26.4)	
Baseline HIV status										
Negative	30.1 (20.3, 40.0)	0.18	14.5 (9.2, 19.9)	<0.01	13.2 (6.8, 19.7)	0.32	3.6 (0.8, 6.5)	0.47	9.6 (5.1, 14.1)	<0.01
Positive	20.8 (13.0, 28.5)		4.0 (0.6, 7.5)		19.3 (10.8, 27.8)		1.6 (0.0, 3.9)		37.7 (29.1, 46.3)	
Initiated PrEP during follow‐up										
No	20.4 (9.1, 31.7)	1.00	14.1 (6.7, 21.5)	1.00	14.3 (4.5, 24.1)	0.78	1.2 (0.0, 3.5)	0.11	7.1 (1.6, 12.7)	0.30
Yes	21.1 (10.5, 31.6)		15.0 (7.2, 22.8)		12.3 (3.8, 20.8)		6.3 (0.9, 11.6)		12.2 (5.1, 19.3)	
Receptive condomless anal intercourse, past three months										
No	17.6 (10.2, 25.0)	0.02	12.3 (7.2, 17.3)	0.02	12.7 (6.3, 19.2)	0.09	3.1 (0.4, 5.7)	0.42	17.1 (11.3, 22.8)	<0.01
Yes	33.8 (22.3, 45.3)		3.2 (0.0, 6.8)		23.1 (12.8, 33.3)		1.1 (0.0, 3.2)		33.3 (23.6, 43.1)	
Number of male partners in past three months										
0 to 2	27.8 (20.2, 35.4)	0.67	10.5 (6.3, 14.6)	0.59	14.3 (8.3, 20.2)	0.30	1.4 (0.0, 3.0)	0.21	20.0 (14.6, 25.4)	0.17
3+	22.9 (8.9, 36.8)		6.5 (0.0, 13.7)		22.9 (8.9, 36.8)		4.4 (0.0, 10.5)		30.4 (17.1, 43.7)	
Any female partners, past 12 months										
No	27.5 (20.4, 34.5)	0.08	9.3 (5.5, 13.0)	0.62	17.0 (11.0, 22.9)	0.30	0.9 (0.0, 2.1)	0.02	23.0 (17.5, 28.5)	0.38
Yes	11.8 (0.9, 22.6)		11.7 (3.5, 19.8)		8.8 (0.0, 18.4)		6.7 (0.4, 13.0)		16.7 (7.2, 26.1)	
Transactional sex, past 12 months										
No	26.6 (19.3, 34.0)	0.01	9.0 (5.2, 12.8)	0.17	17.3 (11.0, 23.5)	0.17	2.3 (0.3, 4.2)	1.00	21.4 (16.1, 26.8)	0.68
Yes	6.3 (0.0, 14.6)		16.3 (5.2, 27.3)		6.3 (0.0, 14.6)		2.3 (0.0, 6.8)		17.1 (5.6, 28.6)	
Injection drug use, past six months										
No	19.0 (7.2, 30.9)	1.00	13.9 (5.9, 21.9)	1.00	11.9 (2.1, 21.7)	1.00	2.8 (0.0, 6.6)	0.25	15.3 (7.0, 23.6)	0.58
Yes	0.0 (0.0, 0.0)		14.3 (0.0, 40.2)		0.0 (0.0, 0.0)		14.3 (0.0, 40.2)		0.0 (0.0, 0.0)	
Any drug use, past six months										
No	26.8 (19.5, 34.0)	0.24	8.7 (4.8, 12.5)	0.19	16.9 (10.7, 23.1)	0.48	1.9 (0.1, 3.8)	0.40	24.6 (18.8, 30.5)	0.05
Yes	17.4 (6.4, 28.3)		13.9 (6.3, 21.6)		10.9 (1.9, 19.9)		3.8 (0.0, 8.0)		13.9 (6.3, 21.6)	
Binge drinking (5 + drinks) on 5 or more days, past 30 days										
No	22.6 (15.6, 29.6)	0.52	12.2 (7.8, 16.6)	0.15	14.6 (8.7, 20.5)	1.00	1.4 (0.0, 3.0)	0.11	20.9 (15.4, 26.3)	0.72
Yes	28.2 (14.1, 42.3)		5.3 (0.0, 11.1)		15.4 (4.1, 26.7)		5.3 (0.0, 11.1)		22.8 (11.9, 33.7)	

Different samples sizes reflect differences in acceptance of screening and missing data. CI, confidence interval; PrEP, pre‐exposure prophylaxis.

^a^ Did not matriculate indicates not completing high school; Matriculate or higher indicates high school graduate or above.

Among 189 participants screened for rectal STI at baseline, 47 (25%) had rectal CT infection and 30 (16%) had rectal NG infection. Among MSM, the prevalence of rectal CT and GC were 24.0% (95% CI: 17.6, 30.4) and 15.2% (95% CI: 9.8, 20.6) respectively. Among TGW, the prevalence of rectal CT and GC were 28.6% (95% CI: 4.9, 52.2) and 21.4% (95% CI: 0.0, 42.9) respectively. Prevalent rectal CT infection was associated in crude estimates with age, sexual identity, receptive condomless anal intercourse in the previous three months, and transactional sex (Table 3). In adjusted models, only age group remained statistically significant, with 18 to 24 year olds having 2.4 (95% CI: 1.1, 5.1) times higher prevalence of rectal CT compared to those 25 and older. Prevalent rectal NG infection was also associated with age in crude and adjusted analyses. Controlling for study site and baseline HIV status, participants age 18 to 24 experienced an incidence rate of rectal NG 3.1 times higher (95% CI: 1.3, 7.1) than those age 25 and older.

Among the 278 participants screened for syphilis at baseline, 50 (18%) had prevalent syphilis infection (21.6% among MSM and 18.2% among TGW). Prevalent syphilis was associated in crude analyses with older age, identifying as gay, being HIV positive and receptive condomless anal intercourse in the past three months.

### 3.2 STI testing and incidence over 12 months of follow‐up

Nearly all (193/201; 96%) participants enrolled in the follow‐up procedures had at least 1 visit where follow‐up STI screening was offered. Of the 193, 144 (75%) accepted rectal screening at least once in follow‐up. Acceptance of at least one urethral (182/193; 94%) and syphilis (181/189; 94%) screening was high during follow‐up. Follow‐up rectal screening was more likely to be accepted among participants who identified as gay compared to some other sexual identity (85.2% vs. 62.8%, *p* < 0.01) and among participants with no female partners in the past 12 months (82.0%) compared to those who did (58.3%, *p* < 0.01). No associations with demographic characteristics remained statistically significant in adjusted models. Urethral and syphilis screening did not significantly differ by study site, participant characteristics or behaviours (Table S1).

The rate of incident urethral CT was 12.8/100 PY and the rate of incident urethral NG was 7.1/ 100 PY. No incident urethral infections were observed among TGW. The incidence of urethral CT was greater among participants in Port Elizabeth (Table 4). This difference persisted in models adjusting for baseline HIV status and age group; the rate of urethral CT was 3.1 (95% CI: 1.2, 8.1) times higher in Port Elizabeth compared to Cape Town. Controlling for study site, age group and baseline HIV status, the incidence rate of urethral NG was 5.1 times higher (95% CI: 1.6, 16.0) among participants reporting transactional sex in the past 12 months.

**Table 4:** Rate (per 100 person years), unadjusted rate ratios (RR), and 95% confidence intervals of urethral and rectal chlamydia, urethral and rectal gonorrhoea, and syphilis among men who have sex with men and transgender women in Cape Town and Port Elizabeth, South Africa

	Chlamydia				Gonorrhea							Syphilis	
	Rectal (N = 127)		Urethral (N = 178)		Rectal (N = 126)		Urethral (N = 179)			(N = 172)			
	Rate (95% CI)	Rate ratio (95% CI)	Rate (95% CI)	Rate ratio (95% CI)	Rate (95% CI)	Rate ratio (95% CI)	Rate (95% CI)		Rate ratio (95% CI)		Rate (95% CI)		Rate ratio (95% CI)
Site													
CapeTown	44.4 (30.0, 65.7)	3.7 (1.3, 10.7)	6.9 (3.1, 15.4)	0.4 (0.1, 0.9)	31.9 (20.1, 50.7)	1.8 (0.7, 4.4)	3.4 (1.1, 10.5)		0.3 (0.1, 1.1)		12.2 (6.6, 22.7)		3.2 (0.9, 11.5)
Port Elizabeth	12.0 (4.5, 31.9)	Ref	19.6 (11.8, 32.5)	Ref	18.1 (8.1, 40.2)	Ref	11.1 (5.8, 21.3)		Ref		3.9 (1.3, 12.0)		Ref
Age ranges													
18‐24	46.0 (30.8, 68.6)	3.4 (1.3, 9.0)	16.1 (9.9, 26.2)	2.1 (0.8, 5.6)	38.3 (24.7, 59.4)	3.6 (1.2, 10.5)	8.8 (4.6, 16.9)		2.0 (0.5, 7.3)		5.9 (2.7, 13.2)		0.5 (0.2, 1.5)
25+	13.3 (5.6, 32.0)	Ref	7.8 (3.3, 18.8)	Ref	10.7 (4.0, 28.5)	Ref	4.5 (1.4, 13.8)		Ref		12.0 (5.7, 25.2)		Ref
Race													
Black	32.0 (21.3, 48.2)	1.0 (0.4, 2.4)	12.8 (8.0, 20.6)	1.0 (0.3, 2.9)	28.0 (18.1, 43.4)	1.3 (0.4, 3.7)	8.0 (4.4, 14.4)		2.5 (0.3, 19.7)		7.7 (4.1, 14.3)		0.7 (0.2, 2.7)
Other	33.4 (15.0, 74.3)	Ref	13.0 (4.9, 34.6)	Ref	22.1 (8.3, 58.8)	Ref	3.1 (0.4, 22.3)		Ref		10.3 (3.3, 31.9)		Ref
Gender identity													
Male	29.7 (19.7, 44.7)	1.0 (0.3, 3.3)	14.6 (9.5, 22.4)	–	19.1 (11.5, 31.7)	0.3 (0.1, 0.8)	8.0 (4.5, 14.0)		–		6.4 (3.3, 12.2)		0.4 (0.1, 2.0)
Transgender or other non‐male identified	30.3 (9.8, 94.1)	Ref	0.0 (0.0, 0.0)	Ref	65.0 (29.2, 144.7)	Ref	0.0 (0.0, 0.0)		Ref		14.6 (3.6, 58.2)		Ref
Sexual identity													
Gay/homosexual	41.2 (27.4, 62.1)	3.3 (1.1, 9.4)	9.3 (4.8, 17.8)	0.5 (0.2, 1.2)	36.2 (23.4, 56.1)	11.9 (1.6, 88.6)	5.0 (2.1, 11.9)		0.5 (0.1, 1.5)		6.3 (2.9, 14.1)		0.8 (0.2, 2.6)
Bisexual, heterosexual, or other	12.7 (4.8, 33.8)	Ref	19.0 (10.8, 33.5)	Ref	3.0 (0.4, 21.6)	Ref	10.6 (5.1, 22.3)		Ref		8.0 (3.3, 19.3)		Ref
Education													
Did notmatriculate^a^	36.6 (22.5, 59.8)	1.6 (0.7, 3.6)	14.7 (8.3, 25.8)	1.3 (0.5, 3.0)	22.1 (11.9, 41.1)	0.8 (0.3, 1.8)	7.0 (3.1, 15.2)		0.9 (0.3, 2.9)		7.5 (3.4, 16.8)		1.0 (0.3, 3.0)
Matriculate or higher	22.7 (12.2, 42.1)	Ref	11.4 (6.0, 22.0)	Ref	28.2 (16.0, 49.6)	Ref	7.4 (3.3, 16.5)		Ref		7.8 (3.5, 17.3)		Ref
Combined work/student													
Part/full‐time student or part/full‐time job	31.4 (19.1, 50.8)	1.0 (0.5, 2.2)	11.7 (6.3, 21.7)	0.8 (0.3, 1.8)	26.8 (15.9, 45.3)	1.2 (0.5, 2.9)	7.9 (3.8, 16.6)		1.2 (0.4, 3.9)		8.3 (4.0, 17.5)		1.5 (0.4, 5.1)
Not a student and no job	30.0 (16.6, 54.2)	Ref	14.9 (8.2, 26.9)	Ref	22.2 (11.1, 44.4)	Ref	6.5 (2.7, 15.5)		Ref		5.5 (2.1, 14.7)		Ref
Income													
No income	45.8 (28.9, 72.7)	2.3 (1.0, 5.0)	14.3 (7.9, 25.9)	1.0 (0.4, 2.5)	24.9 (13.4, 46.2)	1.2 (0.5, 3.0)	6.3 (2.6, 15.1)		0.7 (0.2, 2.2)		6.8 (2.8, 16.3)		1.0 (0.3, 6.4)
Any income	20.2 (10.5, 38.8)	Ref	13.7 (7.4, 25.5)	Ref	20.2 (10.5, 38.8)	Ref	9.2 (4.4, 19.3)		Ref		6.8 (2.9, 16.4)		Ref
Baseline HIV status													
Negative	34.0 (22.8, 50.8)	Ref	12.0 (7.4, 19.6)	Ref	26.8 (17.1, 42.0)	Ref	7.3 (3.9, 13.5)		Ref		7.5 (4.0, 14.0)		Ref
Positive	26.0 (10.8, 62.6)	0.8 (0.3, 2.0)	16.7 (6.9, 40.1)	1.4 (0.5, 3.8)	26.9 (11.2, 64.5)	1.0 (0.4, 2.7)	6.3 (1.6, 25.1)		0.9 (0.2, 3.9)		11.4 (3.7, 35.4)		1.5 (0.4, 5.5)
Initiated PrEP During Follow‐up													
No	30.6 (16.4, 56.8)	Ref	9.5 (4.3, 21.2)	Ref	18.1 (8.2, 40.4)	Ref		9.3 (4.2, 20.7)	Ref		8.0 (3.3, 19.2)		Ref
Yes	37.0 (21.9, 62.5)	1.2 (0.5, 2.7)	14.2 (7.6, 26.4)	1.5 (0.5, 4.1)	34.3 (19.9, 59.0)	1.9 (0.7, 5.0)		5.5 (2.1, 14.5)	0.6 (0.2, 2.1)		7.1 (3.0, 17.0)		0.9 (0.3, 3.1)
Receptive condomless anal intercourse, past three months													
No	21.9 (12.4, 38.5)	Ref	13.3 (7.7, 22.9)	Ref	16.7 (8.7, 32.0)	Ref		7.9 (4.0, 15.8)	Ref		9.5 (5.0, 18.3)		Ref
Yes	57.2 (33.9, 96.6)	2.6 (1.2, 5.6)	8.9 (3.4, 23.8)	0.7 (0.2, 2.1)	47.3 (26.9, 83.4)	2.8 (1.2, 6.7)		2.2 (0.3, 15.5)	0.3 (0.0, 2.2)		7.1 (2.3, 21.9)		0.7 (0.2, 2.7)
Number of male partners in past three months													
0 to 2	31.7 (20.2, 49.7)	Ref	13.9 (8.5, 22.7)	Ref	27.8 (17.3, 44.7)	Ref		7.4 (3.9, 14.3)	Ref		5.2 (2.3, 11.5)		Ref
3+	35.4 (15.9, 78.8)	1.1 (0.4, 2.8)	11.9 (3.8, 36.8)	0.9 (0.2, 2.9)	25.2 (9.4, 67.0)	0.9 (0.3, 2.7)		11.7 (3.8, 36.4)	1.6 (0.2, 5.8)		22.8 (9.5, 54.7)		4.4 (1.4, 14.5)
Any female partners, past 12 months													
No	36.1 (24.5, 52.9)	Ref	12.8 (7.8, 20.9)	Ref	30.6 (20.1, 46.4)	Ref		8.5 (4.7, 15.3)	Ref		8.1 (4.4, 15.1)		Ref
Yes	6.0 (0.8, 42.4)	0.2 (0.0, 1.2)	13.7 (5.7, 32.9)	1.1 (0.4, 2.9)	6.0 (0.9, 42.6)	0.2 (0.0, 1.5)		2.6 (0.4, 18.7)	0.3 (0.0, 2.4)		5.8 (1.5, 23.2)		0.7 (0.2, 3.3)
Transactional sex, past 12 months													
No	35.9 (23.9, 54.0)	Ref	11.5 (6.8, 19.3)	Ref	31.5 (20.3, 48.8)	–		4.7 (2.1, 10.5)	Ref		5.9 (2.8, 12.3)		Ref
Yes	11.9 (3.0, 47.6)	0.3 (0.1, 1.4)	26.4 (12.6, 55.4)	2.3 (0.9, 5.7)	0.0 (0.0, 0.0)	–		21.6 (9.7, 48.1)	4.6 (1.5, 14.2)		14.7 (5.5, 39.1)		2.5 (0.7, 8.5)
Injection drug use, past six months													
No	12.7 (4.1, 39.4)	Ref	17.6 (8.8, 35.1)	Ref	23.0 (9.6, 55.3)	–		10.6 (4.4, 25.5)	Ref		8.5 (3.2, 22.5)		Ref
Yes	26.0 (3.7, 184.3)	2.0 (0.2, 19.6)	19.0 (2.7, 134.6)	1.1 (0.1, 8.6)	0.0 (0.0, 0.0)	–		0.0 (0.0, 0.0)	–		20.6 (2.9, 146.4)		2.4 (0.3, 21.8)
Any drug use, past six months													
No	37.7 (25.1, 56.7)	Ref	10.8 (6.2, 19.1)	Ref	25.4 (15.6, 41.5)	Ref		6.1 (2.9, 12.7)	Ref		6.6 (3.2, 13.9)		Ref
Yes	14.6 (5.5, 38.8)	0.4 (0.1, 1.1)	17.7 (9.2, 34.0)	1.6 (0.7, 3.9)	19.4 (8.1, 46.6)	0.8 (0.3, 2.1)		9.5 (4.0, 22.9)	1.6 (0.5, 4.9)		9.6 (4.0, 23.0)		1.4 (0.5, 4.5)
Binge drinking (5 + drinks) on 5 or more days, past 30 days													
No	27.9 (17.6, 44.3)	Ref	14.8 (9.3, 23.5)	Ref	28.3 (17.8, 44.8)	Ref		7.8 (4.2, 14.5)	Ref		7.5 (3.9, 14.5)		Ref
Yes	31.8 (14.3, 70.7)	1.1 (0.5, 2.9)	6.7 (1.7, 26.8)	0.5 (0.1, 2.0)	20.7 (7.8, 55.1)	0.7 (0.2, 2.2)		3.3 (0.5, 23.6)	0.4 (0.1, 3.3)		7.0 (1.8, 28.1)		0.9 (0.2, 4.3)

CI, confidence interval; PrEP, pre‐exposure prophylaxis.

^a^ Did not matriculate indicates not completing high school; Matriculate or higher indicates high school graduate or above.

The rate of incident rectal CT was 33.4/100 PY and the rate of incident rectal NG was 26.8/100 PY. Rates of rectal CT were similar among MSM (29.7/100 PY) and TGW (30.3/100 PY), but rates of rectal GC were lower among MSM (19.1/100 PY) compared to TGW (65.0/100 PY). The incidence of rectal CT was greater among participants in Cape Town, and those who were aged 18 to 24, identified as gay, reported no income and reported receptive condomless anal intercourse in the past three months. Controlling for study site, baseline HIV status, sexual identity and receptive anal sex in the past three months, being age 18 to 24 (rate ratio (RR) = 2.9, 95% CI: 1.1, 7.7) and reporting no income (RR = 2.5, 95% CI: 1.1, 5.8) were associated with increased rectal CT incidence. The crude rate of rectal NG was greater among participants who were aged 18 to 24, identified as gay, and reported receptive condomless anal intercourse in the past three months. The crude rate of rectal NG was lower among participants who identified as male compared to those with another gender identity. Controlling for study site, baseline HIV status, sexual identity, gender identity and condomless anal sex in the past three months, participants age 18 to 24 experienced a rate of rectal gonorrhoea incidence 5.3 (95% CI: 1.2, 23.7) times higher than those over age 25.

The rate of incident syphilis infection was 8.2/100 PY. Syphilis incidence was higher among TGW (14.6/100 PY) compared to MSM (6.4/100 PY). Syphilis incidence was associated with having 3 or more male partners in the previous three months in crude analyses. This association was no longer statistically significant in a model controlling for study site, age and baseline HIV status.

### 3.3 Symptomatic and concurrent infections

The identification of STI symptoms for infections observed at baseline and follow‐up visits was low. Overall, 91%, 95% and 97% of rectal, urethral and syphilis infections were clinically asymptomatic (Table 5). Of those who received STI testing, 10% had more than one infection concurrently, either one organism at multiple sites or multiple organisms.

**Table 5:** Frequency of urethral NG/CT, rectal NG/CT and syphilis symptoms at baseline, month 6 and month 12 overall and among those with diagnosed STI in a cohort of men who have sex with men and transgender women in Cape Town and Port Elizabeth, South Africa

	Baseline – all	Baseline – STI + ^a^	Month 6	Month 6 – STI + ^a^	Month 12	Month 12 – STI + ^a^
	n/N (%)	n/N (%)	n/N (%)	n/N (%)	n/N (%)	n/N (%)
Urethral STI symptoms	3/292 (1.0%)	1/34 (2.9%)	1/172 (0.6%)	0/12 (0.0%)	2/174 (1.2%)	1/15 (6.7%)
Rectal STI symptoms	6/292 (2.0%)	5/60 (8.3%)	0/172 (0.0%)	0/24 (0.0%)	3/174 (1.7%)	3/26 (11.5%)
Syphilis symptoms	4/292 (1.4%)	1/50 (2.0%)	5/172 (2.9%)	1/16 (6.3%)	1/174 (0.6%)	1/22 (4.6%)

All enrolled participants contributed to baseline data; all HIV negative and a sample of HIV‐positive participants were prospectively followed and contributed data at months 6 and 12. CT, *Chlamydia trachomatis*; NG, *Neisseria gonorrhea*; STI, sexually transmitted infections.

^a^ The denominator for each cell is the number of participants diagnosed with a relevant STI (e.g. urethral NG or CT for those with urethral symptoms).

### 3.4 PrEP use

There were no differences in the incidence of CT, NG or syphilis among participants who initiated PrEP during study follow‐up compared to those who did not.

## 4 DISCUSSION

We implemented a comprehensive package of HIV/STI screening and treatment with high acceptance among MSM and TGW in South Africa. Our study population was comprised of a baseline cohort of whom all HIV negative and a sample of HIV‐positive participants were prospectively followed for one year. Urethral STI and syphilis screening were high overall, but rectal screening acceptance was substantially lower in Port Elizabeth compared to Cape Town at baseline and during follow‐up. We observed exceptionally high prevalence and incidence of rectal STIs, the vast majority of which were asymptomatic, consistent with previous findings among MSM [18]. Because the prevalence of rectal infections was higher than urethral infections, this difference in willingness to screen has important implications for the STI epidemics in each city. The current STI management guidelines in South Africa, adapted from the World Health Organization, call for syndromic management of STIs [6]. Given the high prevalence of asymptomatic STIs in our study population, it is likely that a syndromic approach is inadequate to detect STIs among MSM and TGW. This study was conducted from 2015 to 2016; however, the environment with respect to STI incidence and prevalence has been stable for decades [19], and we believe these data remain relevant. The continuing reliance on syndromic management will result in many missed opportunities to identify and treat infections compared to screening.

The prevalence of CT, NG and syphilis was high in both study sites. The prevalence of rectal CT and NG was substantially higher than the prevalence of urethral infection, similar to previous studies [18, 20]. In both cities, more than one‐fifth of participants had a rectal STI at baseline. Approximately 20% of the study population had syphilis at the baseline visit. These findings represent substantial unmet needs for STI screening and treatment among MSM and TGW in these South African cities. Younger participants were more likely to have rectal NG/CT, and participants who identified as gay were more likely to have rectal CT at the baseline visit. Participants who reported receptive condomless anal sex in the previous three months had higher prevalence of rectal and other STIs. Previous studies have found similar characteristics to be associated with asymptomatic NG/CT infection including transgender identity, multiple male sex partners in the previous 12 months and transactional sex [13].

Incident STIs followed a similar pattern. The incidence of rectal infection was higher than urethral infection at both study sites for both CT and NG. Higher incidence rates of rectal infections and syphilis were observed in Cape Town compared to Port Elizabeth; however, the difference was only statistically significant for rectal CT. The lower acceptance of rectal STI screening in Port Elizabeth compared to Cape Town (72.5% vs. 83.0%) might at least partially account for the difference in rectal CT incidence. The acceptance of syphilis screening and urethral STI screening was near universal at both sites, so we believe that all or most incident urethral and syphilis infections were detected; however, some rectal STIs might have been missed due to lower acceptance of rectal screening. It remains unclear what led some participants to refuse rectal screening. It might be the case that those at the highest risk of rectal infections were more likely to accept rectal screening; however, there were no differences in rectal screening acceptance based on reporting anal intercourse in the past three months. A recent study of Thai TGW found that rectal screening produced the highest yield of positive NG/CT infections [10], implying that rectal screening will be vitally important to reduce NG/CT incidence and prevalence. Future studies should assess reasons for refusal of rectal screening. We did not observe differences in STI incidence based on gender identity. However, we did not observe any incident urethral infections among TGW. The rate of rectal GC and syphilis were much higher among TGW compared to MSM; however, the CI for these rates were very wide due to the small sample size of TGW and the differences were not statistically significant. We did not screen for pharyngeal infection. However, there is evidence that pharyngeal NG/CT infections can cause urethral [21] and rectal [22] infections in sexual partners. Therefore, it remains necessary to characterize the burden of pharyngeal infections among MSM and TGW in South Africa and pharyngeal screening should be part of all STI screening programmes.

Age, sexual identity and condomless receptive anal sex were all associated with incident infection, consistent with the associations observed for prevalent infections at baseline. These characteristics might be useful in identifying MSM and TGW in need of more frequent STI screening due to increased risk, and align with findings from other studies of incident STI among MSM [14]. Indeed, the WHO guidelines for prevention and treatment of STIs among MSM and TGW [23] call for presumptive treatment of STIs among MSM and TGW who report receptive anal intercourse and either multiple partners or a partner with a STI in the past six months; unfortunately, these guidelines do not include recommendations on how frequently presumptive treatment should occur. Our data support the WHO guidelines, however, implementation of these guidelines in the absence of screening will still result in missing substantial numbers of asymptomatic infections [24]. Based on our data, a large proportion of STIs are asymptomatic, a phenomenon observed elsewhere [9]. In the absence of screening, individuals would not be able to report a partner with an asymptomatic STI. It is unlikely that presumptive treatment will be sufficient to meaningfully reduce the STI burden in these key populations. Rather, incorporation of point‐of‐care screening [25] to diagnose both symptomatic and asymptomatic STIs will likely have a greater effect on the STI epidemic among MSM and TGW.

There is growing interest in the intersection of HIV and other STIs [26]. A recent modelling study estimated that approximately 10% of HIV incidence among MSM might be attributable to prevalent NG and CT [27], suggesting that STI detection and treatment might lead to meaningful reductions in HIV incidence. Additionally, there are concerns that MSM and TGW who use HIV pre‐exposure prophylaxis (PrEP) may continue to have (or increase frequency of) condomless anal sex once PrEP has been started, a phenomenon known as risk compensation [28]. Although some studies have observed little or no behavioural risk compensation [15, 28, 29], a recent review found an increased risk of rectal CT among PrEP‐using MSM and TGW [30]. Condomless anal sex may lead to STI acquisition, which could also undermine the HIV prevention benefits of PrEP by increasing biological risks for HIV infection. We did not observe differences in STI incidence between PrEP users and non‐users in this study. Surveillance estimates indicate HIV prevalence is higher than 18% among MSM in South Africa [31], yet STI prevalence among MSM is unreported and there are no previous studies examining STI incidence and rectal STI screening in this group.

This study has a number of limitations. First, these data were generated as part of a pilot study of a combination HIV prevention package that was not specifically powered to examine STI prevalence and incidence and associated risk factors. Thus, our estimates are imprecise; the direction and relative strength of the observed associations should be used to generate hypotheses that can be tested in larger studies. As described earlier, we conducted RPR and confirmatory TPPA syphilis testing and monitored titres over the course of the study; however, it is possible that some of the prevalent syphilis infections at baseline had previously been treated. Some participants were referred to community clinics for STI treatment, and completion of a treatment regimen could not be verified. Low acceptance of rectal STI screening indicates that our estimates of rectal STI prevalence and incidence are underestimates and could be prone to information bias.

A major limitation of this study is the small sample of TGW who were enrolled: We are limited in our ability to make inferences about predictors of prevalence and incidence among TGW specifically and about differences between MSM and TGW due to the small sample of TGW enrolled in the study. We are also unable to assess differences between TGW who choose to participate in a study that primarily comprises MSM. MSM and TGW are both marginalized populations who frequently experience stigma in healthcare settings [32]; however, they are also unique identities and each group has unique needs. Future studies should make efforts to focus specifically and more robustly on the needs and experiences of TGW with respect to STI screening, incidence and prevalence.

## 5 CONCLUSIONS

STIs are highly prevalent among MSM and TGW in South Africa, and rectal STIs are more common than urethral infections. High incidence rates indicate ongoing STI risk even following successful treatment. Because the vast majority of STIs in our study were asymptomatic, multi‐site STI screening and treatment among MSM and TGW are of paramount importance in combating the STI epidemic.

## COMPETING INTERESTS

The authors have no conflicts to disclose.

## AUTHORS’ CONTRIBUTIONS

TS, LGB, NPM, SB, AM, AJS and PSS contributed to study design; KD, LK, CY and RZ contributed to data collection; JJ, TS and RV conducted the analyses; JJ and TS drafted the manuscript; all authors read and approved the final version of the manuscript.

## Supporting information


**Table S1.** Acceptance of urethral and syphilis STI screening at baseline and over 12 months of follow‐up among men who have sex with men and transgender women in Cape Town and Port Elizabeth, South Africa
**Table S2.** Rate (per 100 person years), unadjusted rate ratios (RR), and 95% confidence intervals of urethral and rectal chlamydia, urethral and rectal gonorrhea, and syphilis among men who have sex with men (MSM) in Cape Town and Port Elizabeth, South Africa

## References

[1] World Health Organization . Report on global sexually transmitted infection surveillance 2015. Geneva, Switzerland: World Health Organization; 2016.

[2] Centers for Disease Control and Prevention . Sexually transmitted disease surveillance 2016. Atlanta, GA: U.S. Department of Health and Human Services; 2017.

[3] The South African National AIDS Council . South Africa's National strategic plan for HIV, TB, and STIs 2017‐2022. 2017.

[4] Farley TA , Cohen DA , Elkins W . Asymptomatic sexually transmitted diseases: the case for screening. Prev Med. 2003;36(4):502–9. 12649059 10.1016/s0091-7435(02)00058-0

[5] Marcus JL , Bernstein KT , Kohn RP , Liska S , Philip SS . Infections missed by urethral‐only screening for chlamydia or gonorrhea detection among men who have sex with men. Sex Transm Dis. 2011;38(10):922–4. 21934565 10.1097/OLQ.0b013e31822a2b2e

[6] Department of Health Republic of South Africa . Sexually transmitted infections management guidelines 2015. 2015.

[7] Mustanski B , Feinstein BA , Madkins K , Sullivan P , Swann G . Prevalence and risk factors for rectal and urethral sexually transmitted infections from self‐collected samples among young men who have sex with men participating in the keep it up! 2.0 randomized controlled trial. Sex Transm Dis. 2017;44(8):483–88. 28703727 10.1097/OLQ.0000000000000636 PMC5821498

[8] Yang LG , Zhang XH , Zhao PZ , Chen Z‐Y , Ke W‐J , Ren X‐Q , et al. Gonorrhea and chlamydia prevalence in different anatomical sites among men who have sex with men: a cross‐sectional study in Guangzhou, China. BMC Infect Dis. 2018;18:675. 30563478 10.1186/s12879-018-3579-6 PMC6299533

[9] Keshinro B , Crowell TA , Nowak RG , Adebajo S , Peel S , Gaydos CA , et al. High prevalence of HIV, chlamydia and gonorrhoea among men who have sex with men and transgender women attending trusted community centres in Abuja and Lagos, Nigeria. J Int AIDS Soc. 2016;19:21270. 27931519 10.7448/IAS.19.1.21270 PMC5146323

[10] Hiransuthikul A , Janamnuaysook R , Sungsing T , Callander D , Fairley CK , Grulich AE , et al. High burden of chlamydia and gonorrhoea in pharyngeal, rectal and urethral sites among Thai transgender women: implications for anatomical site selection for the screening of STI. Sex Transm Infect. 2019;95(7):534–9. 30982000 10.1136/sextrans-2018-053835

[11] Comninos NB , Garton L , Guy R , Callander D , Fairley CK , Grulich AE , et al. Increases in pharyngeal Neisseria gonorrhoeae positivity in men who have sex with men, 2011‐2015: observational study. Sex Transm Infect. 2019. 10.1136/sextrans-2019-054107 31624177

[12] Sullivan PS , Peterson J , Rosenberg ES , Kelley CF , Cooper H , Vaughan A , et al. Understanding racial HIV/STI disparities in black and white men who have sex with men: a multilevel approach. PLoS One. 2014;9:e90514. 24608176 10.1371/journal.pone.0090514 PMC3946498

[13] Rebe K , Lewis D , Myer L , de Swardt G , Struthers H , Kamkuemah M , et al. A cross sectional analysis of gonococcal and chlamydial infections among men‐who‐have‐sex‐with‐men in Cape Town, South Africa. PLoS One. 2015;10:e0138315. 26418464 10.1371/journal.pone.0138315 PMC4587970

[14] Ramadhani HO , Liu H , Nowak RG , Crowell TA , Ndomb T , Gaydos C , et al. Sexual partner characteristics and incident rectal Neisseria gonorrhoeae and *Chlamydia trachomatis* infections among gay men and other men who have sex with men (MSM): a prospective cohort in Abuja and Lagos, Nigeria. Sex Transm Infect. 2017;93(5):348–55. 28235839 10.1136/sextrans-2016-052798 PMC5527124

[15] Hightow‐Weidman LB , Magnus M , Beauchamp G , Hurt CB , Shoptaw S , Emel L , et al. Incidence and correlates of STIs among black men who have sex with men participating in the HPTN 073 PrEP study. Clin Infect Dis. 2019;69(9):1597‐604. 30615169 10.1093/cid/ciy1141 PMC6792108

[16] McNaghten A , Kearns R , Siegler AJ , Phaswana‐Mafuya N , Bekker L‐G , Stephenson R , et al. Sibanye methods for prevention packages program project protocol: pilot study of HIV prevention interventions for men who have sex with men in South Africa. JMIR Res Protoc. 2014;3(4):e55. 10.2196/resprot.3737 PMC4210958 25325296

[17] Spiegelman D , Hertzmark E . Easy SAS calculations for risk or prevalence ratios and differences. Am J Epidemiol. 2005;162(3):199–200. 15987728 10.1093/aje/kwi188

[18] Annan NT , Sullivan AK , Nori A , Naydenova P , Alexander S , McKenna A , et al. Rectal chlamydia—a reservoir of undiagnosed infection in men who have sex with men. Sex Transm Infect. 2009;85(3):176–9. 19176570 10.1136/sti.2008.031773

[19] Kularatne RS , Niit R , Rowley J , Kufa‐Chakezha T , Peters RPH , Taylor MM , et al. Adult gonorrhea, chlamydia and syphilis prevalence, incidence, treatment and syndromic case reporting in South Africa: estimates using the spectrum‐STI model, 1990–2017. PLoS One. 2018;13:e0205863. 30321236 10.1371/journal.pone.0205863 PMC6188893

[20] Kent CK , Chaw JK , Wong W , Liska S , Gibson S , Hubbard G , et al. Prevalence of rectal, urethral, and pharyngeal chlamydia and gonorrhea detected in 2 clinical settings among men who have sex with men: San Francisco, California, 2003. Clin Infect Dis. 2005;41(1):67–74. 15937765 10.1086/430704

[21] Bernstein KT , Stephens SC , Barry PM , Kohn R , Philip SS , Liska S , et al. *Chlamydia trachomatis* and Neisseria gonorrhoeae transmission from the oropharynx to the urethra among men who have sex with men. Clin Infect Dis. 2009;49(12):1793–7. 19911970 10.1086/648427

[22] McMillan A , Young H , Moyes A . Rectal gonorrhoea in homosexual men: source of infection. Int J STD AIDS. 2000;11(5):284–7. 10824935 10.1177/095646240001100502

[23] World Health Organization . Guidelines: prevention and treatment of HIV and other sexually transmitted infections among men who have sex with men and transgender people. Geneva: World Health Organization; 2011. 26158187

[24] Sanders EJ , Wahome E , Okuku HS , Thiong'o AN , Smith AD , Duncan S , et al. Evaluation of WHO screening algorithm for the presumptive treatment of asymptomatic rectal gonorrhoea and chlamydia infections in at‐risk MSM in Kenya. Sex Transm Infect. 2014;90(2):94–9. 24327758 10.1136/sextrans-2013-051078 PMC3932748

[25] Tucker JD , Bien CH , Peeling RW . Point‐of‐care testing for sexually transmitted infections: recent advances and implications for disease control. Curr Opin Infect Dis. 2013;26(1):73–9. 23242343 10.1097/QCO.0b013e32835c21b0 PMC3635142

[26] Cohen MS , Council OD , Chen JS . Sexually transmitted infections and HIV in the era of antiretroviral treatment and prevention: the biologic basis for epidemiologic synergy. J Int AIDS Soc. 2019;22:e25355. 31468737 10.1002/jia2.25355 PMC6715951

[27] Jones J , Weiss K , Mermin J , Dietz P , Rosenberg ES , Gift TL , et al. Proportion of incident HIV cases among men who have sex with men attributable to gonorrhea and chlamydia: a modeling analysis. Sex Transm Dis. 2019;46(6):357–63. 31095100 10.1097/OLQ.0000000000000980 PMC6530490

[28] Freeborn K , Portillo CJ . Does pre‐exposure prophylaxis for HIV prevention in men who have sex with men change risk behaviour? A systematic review. J Clin Nurs. 2018;27(17–18):3254–65. 28771856 10.1111/jocn.13990 PMC5797507

[29] Milam J , Jain S , Dube MP , Daar ES , Sun X , Corado K , et al. Sexual risk compensation in a pre‐exposure prophylaxis demonstration study among individuals at risk for HIV. J Acquir Immune Defic Syndr. 2019;80(1):e9–e13. 10.1097/QAI.0000000000001885 PMC6289757 30334877

[30] Traeger MW , Schroeder SE , Wright EJ , Hellard ME , Cornelisse VJ , Doyle JS , et al. Effects of pre‐exposure prophylaxis for the prevention of human immunodeficiency virus infection on sexual risk behavior in men who have sex with men: a systematic review and meta‐analysis. Clin Infect Dis. 2018;67(5):676–86. 29509889 10.1093/cid/ciy182

[31] UNAIDS . AIDSinfo. UNAIDS 2019 [cited 2019 Sep 2]. Available from: http://aidsinfo.unaids.org/

[32] Lyons C , Stahlman S , Holland C , Ketende S , Van Lith L , Kochelani D , et al. Stigma and outness about sexual behaviors among cisgender men who have sex with men and transgender women in Eswatini: a latent class analysis. BMC Infect Dis. 2019;19(1):211. 30832602 10.1186/s12879-019-3711-2 PMC6399954

