# Supplementary material for: Sexually transmitted infection screening, prevalence and incidence among South African men and transgender women who have sex with men enrolled in a combination HIV prevention cohort study: the Sibanye Methods for Prevention Packages Programme (MP3) project
Source: J Int AIDS Soc. 2020 Oct 1;23(Suppl 6):e25594. doi: 10.1002/jia2.25594 (PMC7527766; doi:10.1002/jia2.25594)
Supplement: Supplementary file 1 — Table S1. Acceptance of urethral and syphilis STI screening at baseline and over 12 months of follow‐up among men who have sex with men and transgender women in Cape Town and Port Elizabeth, South Africa Table S2. Rate (per 100 person years), unadjusted rate ratios (RR), and 95% confidence intervals of urethral and rectal chlamydia, urethral and rectal gonorrhea, and syphilis among men who have sex with men (MSM) in Cape Town and Port Elizabeth, South Africa [file JIA2-23-e25594-s001.docx]

**Appendix**

Supplemental Table 1. Acceptance of urethral and syphilis STI screening at baseline and over 12 months of follow-up among men who have sex with men and transgender women in Cape Town and Port Elizabeth, South Africa. All participants (N = 292) are included in the baseline estimates; only prospective participants (N = 201) are included in the follow-up estimates. Prospective participants are all HIV-negative participants and a sample of HIV-positive participants.

|  | Urethral STI Screening | | | | | | | | Syphilis | | | | | | | | |
| --- | --- | --- | --- | --- | --- | --- | --- | --- | --- | --- | --- | --- | --- | --- | --- | --- | --- |
|  | Baseline  (N=292) | | | Follow-up  (N=189) | | | Baseline  (N=292) | | | | | | Follow-up  (N=189) | | | |  |
|  | Prevalence  (95% CI) | p-value | | Prevalence  (95% CI) | p-value | | Prevalence  (95% CI) | | | | p-value | | Prevalence  (95% CI) | | p-value | |  |
| **Site** |  |  | |  |  | |  | | | |  | |  | |  | |  |
| Cape Town | 100.0 (100.0, 100.0) | - | | 95.7 (91.7, 99.8) | 0.72 | | 100.0 (100.0, 100.0) | | | | 0.28 | | 95.7 (91.7, 99.8) | | 1.00 | |  |
| Port Elizabeth | 100.0 (100.0, 100.0) |  | | 96.8 (93.3, 100.0) |  | | 98.3 (96.4, 100.0) | | | |  | | 95.8 (91.8, 99.8) | |  | |  |
| **Age ranges** |  |  | |  |  | |  | | | |  | |  | |  | |  |
| 18-24 | 100.0 (100.0, 100.0) | - | | 96.5 (93.2, 99.9) | 1.00 | | 98.2 (96.1, 100.0) | | | | 0.26 | | 95.7 (91.9, 99.4) | | 1.00 | |  |
| 25+ | 100.0 (100.0, 100.0) |  | | 95.9 (91.5, 100.0) |  | | 100.0 (100.0, 100.0) | | | |  | | 95.9 (91.5, 100.0) | |  | |  |
| **Race** |  |  | |  |  | |  | | | |  | |  | |  | |  |
| Black | 100.0 (100.0, 100.0) | - | | 96.1 (93.0, 99.2) | 1.00 | | 98.8 (97.5, 100.0) | | | | 1.00 | | 95.5 (92.2, 98.7) | | 1.00 | |  |
| Other | 100.0 (100.0, 100.0) |  | | 97.1 (91.6, 100.0) |  | | 100.0 (100.0, 100.0) | | | |  | | 97.1 (91.6, 100.0) | |  | |  |
| **Gender Identity** |  |  | |  |  | |  | | | |  | |  | |  | |  |
| Male | 100.0 (100.0, 100.0) | - | | 95.9 (92.9, 98.9) | 1.00 | | 98.9 (97.6, 100.0) | | | | 1.00 | | 95.3 (92.1, 98.5) | | 1.00 | |  |
| Transgender or other non-male identified | 100.0 (100.0, 100.0) |  | | 100.0 (100.0, 100.0) |  | | 100.0 (100.0, 100.0) | | | |  | | 100.0 (100.0, 100.0) | |  | |  |
| **Sexual Identity** |  |  | |  |  | |  | | | |  | |  | |  | |  |
| Gay/homosexual | 100.0 (100.0, 100.0) | - | | 97.2 (94.1, 100.0) | 0.46 | | 99.0 (97.5, 100.0) | | | | 1.00 | | 97.2 (94.1, 100.0) | | 0.29 | |  |
| Bisexual, heterosexual, or other | 100.0 (100.0, 100.0) |  | | 94.9 (90.0, 99.8) |  | | 100.0 (100.0, 100.0) | | | |  | | 93.6 (88.2, 99.0) | |  | |  |
| **Education** |  |  | |  |  | |  | | | |  | |  | |  | |  |
| Did not matriculate | 100.0 (100.0, 100.0) | - | | 96.8 (93.2, 100.0) | 1.00 | | 100.0 (100.0, 100.0) | | | | 0.25 | | 96.8 (93.2, 100.0) | | 0.72 | |  |
| Matriculate or Higher | 100.0 (100.0, 100.0) |  | | 95.7 (91.6, 99.8) |  | | 98.0 (95.8, 100.0) | | | |  | | 94.6 (90.0, 99.2) | |  | |  |
| **Combined work/student** |  |  | |  |  | |  | | | |  | |  | |  | |  |
| Part/full-time student or part/full-time job | 100.0 (100.0, 100.0) | - | | 96.9 (93.4, 100.0) | 0.71 | | 98.7 (96.8, 100.0) | | | | 1.00 | | 96.9 (93.4, 100.0) | | 0.48 | |  |
| Not a student and no job | 100.0 (100.0, 100.0) |  | | 95.5 (91.2, 99.8) |  | | 99.3 (97.8, 100.0) | | | |  | | 94.4 (89.6, 99.2) | |  | |  |
| **Income** |  |  | |  |  | |  | | | |  | |  | |  | |  |
| No income | 100.0 (100.0, 100.0) | - | | 94.4 (89.7, 99.2) | 0.44 | | 99.3 (97.9, 100.0) | | | | 0.61 | | 94.4 (89.7, 99.2) | | 0.72 | |  |
| Any Income | 100.0 (100.0, 100.0) |  | | 97.6 (94.4, 100.0) |  | | 98.5 (96.3, 100.0) | | | |  | | 96.5 (92.5, 100.0) | |  | |  |
| **Baseline HIV Status** |  |  | |  |  | |  | | | |  | |  | |  | |  |
| Negative | 100.0 (100.0, 100.0) | - | | 96.8 (94.0, 99.6) | 0.35 | | 99.4 (98.2, 100.0) | | | | 0.58 | | 96.2 (93.1, 99.2) | | 0.63 | |  |
| Positive | 100.0 (100.0, 100.0) |  | | 93.9 (85.8, 100.0) |  | | 98.4 (96.2, 100.0) | | | |  | | 93.9 (85.8, 100.0) | |  | |  |
| **Initiated PrEP During Follow-up** | | |  |  |  |  | | | |  | |  | |  | |  |  |
| No | 100.0 (100.0, 100.0) | - | | 93.4 (87.8, 99.0) | 0.03 | | | 98.8 (96.5, 100.0) | | | 1.00 | | 92.1 (86.0, 98.2) | | 0.01 | |  |
| Yes | 100.0 (100.0, 100.0) |  | | 100.0 (100.0, 100.0) |  | | | 100.0 (100.0, 100.0) | | |  | | 100.0 (100.0, 100.0) | |  | |  |
| **Receptive condomless anal intercourse, past 3 months** | | | |  |  |  | | | |  | |  | |  | |  |  |
| No | 100.0 (100.0, 100.0) | - | | 95.7 (92.0, 99.4) | 1.00 | | | 99.4 (98.2, 100.0) | | | 0.30 | | 94.8 (90.8, 98.9) | | 1.00 | |  |
| Yes | 100.0 (100.0, 100.0) |  | | 95.8 (90.2, 100.0) |  | | | 97.8 (94.9, 100.0) | | |  | | 95.8 (90.2, 100.0) | |  | |  |
| **Number of male partners in past 3 months** | | |  |  |  |  | | | |  | |  | |  | |  |  |
| 0-2 | 100.0 (100.0, 100.0) | - | | 97.0 (94.1, 99.9) | 1.00 | | | 99.1 (97.8, 100.0) | | | 1.00 | | 96.2 (93.0, 99.5) | | 1.00 | |  |
| 3+ | 100.0 (100.0, 100.0) |  | | 96.4 (89.6, 100.0) |  | | | 100.0 (100.0, 100.0) | | |  | | 96.4 (89.6, 100.0) | |  | |  |
| **Any female partners, past 12 months** | | |  |  |  |  | | | |  | |  | |  | |  |  |
| No | 100.0 (100.0, 100.0) | - | | 98.6 (96.6, 100.0) | 0.01 | | | 98.7 (97.2, 100.0) | | | 1.00 | | 98.6 (96.6, 100.0) | | <0.01 | |  |
| Yes | 100.0 (100.0, 100.0) |  | | 89.6 (80.9, 98.2) |  | | | 100.0 (100.0, 100.0) | | |  | | 87.5 (78.1, 96.9) | |  | |  |
| **Transactional sex, past 12 months** | | |  |  |  |  | | | |  | |  | |  | |  |  |
| No | 100.0 (100.0, 100.0) | - | | 97.1 (94.3, 99.9) | 0.34 | | | 99.1 (97.9, 100.0) | | | 1.00 | | 96.4 (93.3, 99.5) | | 0.63 | |  |
| Yes | 100.0 (100.0, 100.0) |  | | 94.1 (86.2, 100.0) |  | | | 100.0 (100.0, 100.0) | | |  | | 94.1 (86.2, 100.0) | |  | |  |
| **Injection drug use, past 6 months** | | |  |  |  |  | | | |  | |  | |  | |  |  |
| No | 100.0 (100.0, 100.0) | - | | 96.4 (91.4, 100.0) | 1.00 | | | 100.0 (100.0, 100.0) | | | -- | | 96.4 (91.4, 100.0) | | 1.00 | |  |
| Yes | 100.0 (100.0, 100.0) |  | | 100.0 (100.0, 100.0) |  | | | 100.0 (100.0, 100.0) | | |  | | 100.0 (100.0, 100.0) | |  | |  |
| **Any drug use, past 6 months** |  |  | |  |  | | |  | | |  | |  | |  | |  |
| No | 100.0 (100.0, 100.0) | - | | 96.0 (92.6, 99.4) | 1.00 | | | 98.6 (97.0, 100.0) | | | 0.56 | | 95.2 (91.5, 99.0) | | 1.00 | |  |
| Yes | 100.0 (100.0, 100.0) |  | | 96.7 (92.3, 100.0) |  | | | 100.0 (100.0, 100.0) | | |  | | 96.7 (92.3, 100.0) | |  | |  |
| **Binge drinking (5+ drinks) on 5 or more days, past 30 days** | | | |  |  |  | | | |  | |  | |  | |  |  |
| No | 100.0 (100.0, 100.0) | - | | 97.1 (94.4, 99.9) | 0.34 | | | 99.1 (97.8, 100.0) | | | 0.51 | | 96.4 (93.4, 99.5) | | 0.63 | |  |
| Yes | 100.0 (100.0, 100.0) |  | | 94.3 (86.6, 100.0) |  | | | 98.2 (94.8, 100.0) | | |  | | 94.3 (86.6, 100.0) | |  | |  |

Caption: PrEP = pre-exposure prophylaxis; *Did not matriculate indicates not completing high school; Matriculate or higher indicates high school graduate or above

Supplemental Table 2. Rate (per 100 person years), unadjusted rate ratios (RR), and 95% confidence intervals of urethral and rectal chlamydia, urethral and rectal gonorrhea, and syphilis among men who have sex with men (MSM) in Cape Town and Port Elizabeth, South Africa. MSM and transgender women are distinct populations. The study population was predominantly MSM, so we present these results to allow comparisons of the overall study population and MSM only.

|  | Chlamydia | | | | | Gonorrhea | | | | | | | Syphilis | | | | | |  |
| --- | --- | --- | --- | --- | --- | --- | --- | --- | --- | --- | --- | --- | --- | --- | --- | --- | --- | --- | --- |
|  | Rectal  (N=109) | | | Urethral  (N=159) | | | Rectal  (N=108) | | | Urethral  (N=160) | | | | (N=153) | |  | | | |
|  | Rate | | RR  (95% CI) | Rate | RR  (95% CI) | | | Rate | RR  (95% CI) | | Rate | RR  (95% CI) | | | Rate | | RR  (95% CI) |  |  |
| **Site** |  | |  |  |  | | |  |  | |  |  | | |  | |  |  |  |
| Cape Town | 43.5  (28.1, 67.4) | | 4.6  (1.4, 15.4) | 8.3  (3.7, 18.4) | 0.4  (0.2, 1.0) | | | 21.2  (11.4, 39.4) | 1.3  (0.5, 3.9) | | 4.0  (1.3, 12.5) | 0.3  (0.1, 1.3) | | | 8.7  (3.9, 19.4) | | 2.1  (0.5, 8.4) |  |  |
| Port Elizabeth | 9.5  (3.1, 29.5) | | Ref | 20.9  (12.6, 34.7) | Ref | | | 16.0  (6.7, 38.5) | Ref | | 11.8  (6.2, 22.7) | Ref | | | 4.1  (1.3, 12.8) | | Ref |  |  |
| **Age ranges** |  | |  |  |  | | |  |  | |  |  | | |  | |  |  |  |
| 18-24 | 40.0  (25.2, 63.4) | | 2.6  (1.0, 7.0) | 18.8  (11.5, 30.6) | 2.2  (0.8, 6.0) | | | 28.8  (16.7, 49.6) | 4.8  (1.1, 21.2) | | 10.2  (5.3, 19.0) | 2.1  (0.6, 7.8) | | | 4.6  (1.7, 12.2) | | 0.5  (0.1, 1.8) |  |  |
| 25+ | 15.4  (6.4, 37.0) | | Ref | 8.5  (3.5, 20.4) | Ref | | | 6.0  (1.5, 24.0) | Ref | | 4.8  (1.6, 15.0) | Ref | | | 9.3  (3.9, 22.3) | | Ref |  |  |
| **Race** |  | |  |  |  | | |  |  | |  |  | | |  | |  |  |  |
| Black | 28.2  (17.8, 44.8) | | 0.8  (0.3, 2.1) | 14.3  (8.9, 22.9) | 0.9  (0.3, 2.6) | | | 23.5  (14.2, 38.9) | -- | | 8.8  (4.9, 15.9) | 2.3  (0.3, 17.7) | | | 6.8  (3.4, 13.6) | | 1.6  (0.2, 13.0) |  |  |
| Other | 36.5  (15.2, 87.7) | | Ref | 16.1  (6.0, 42.9) | Ref | | | 0.0  (--) | Ref | | 3.9  (0.5, 27.4) | Ref | | | 4.2  (0.6, 29.8) | | Ref |  |  |
| **Sexual Identity** |  | |  |  |  | | |  |  | |  |  | | |  | |  |  |  |
| Gay/homosexual | 40.7  (26.0, 63.8) | | 3.0  (1.0, 8.9) | 10.6  (5.5, 20.4) | 0.5  (0.2, 1.2) | | | 27.8  (16.1, 47.9) | 8.6  (1.1, 66.1) | | 5.7  (2.4, 10.0) | 0.5  (0.2, 1.6) | | | 4.8  (1.8, 12.9) | | 0.6  (0.1, 2.1) |  |  |
| Bisexual or Other | 13.4  (5.0, 35.8) | | Ref | 20.6  (11.7, 36.3) | Ref | | | 3.2  (0.5, 22.8) | Ref | | 11.5  (5.5, 24.1) | Ref | | | 8.7  (3.6, 20.9) | | Ref |  |  |
| **Circumcision** |  | |  |  |  | | |  |  | |  |  | | |  | |  |  |  |
| Full or Partial | 19.8  (10.3, 38.0) | | Ref | 10.3  (4.9, 21.6) | Ref | | | 13.0  (5.8, 28.9) | Ref | | 7.1  (3.0, 17.1) | Ref | | | 0.0  (--) | | Ref |  |  |
| Uncircumcised | 52.7  (31.2, 89.0) | | 2.7  (1.2, 6.2) | 14.7  (6.6, 32.8) | 1.4  (0.5, 4.3) | | | 26.3  (12.5, 55.1) | 2.0  (0.7, 6.0) | | 4.7  (1.2, 18.9) | 0.7  (0.1, 3.4) | | | 18.4  (8.8, 38.5) | | -- |  |  |
| No exam | 0.0  (--) | | -- | 22.7  (11.4, 45.4) | 2.2  (0.8, 6.1) | | | 35.9  (9.0, 143.5) | 2.8  (0.6, 13.7) | | 13.2  (5.5, 31.7) | 1.9  (0.5, 6.4) | | | 5.2  (1.3, 20.8) | | -- |  |  |
| **Education** |  | |  |  |  | | |  |  | |  |  | | |  | |  |  |  |
| Did not matric | 39.4  (23.3, 66.5) | | 2.0  (0.8, 4.7) | 17.3  (9.8, 30.4) | 1.4  (0.6, 3.3) | | | 10.4  (3.9, 27.6) | 0.4  (0.1, 1.3) | | 8.2  (3.7, 18.2) | 1.0  (0.3, 3.2) | | | 4.4  (1.4, 13.6) | | 0.5  (0.1, 2.1) |  |  |
| Matric or Higher | 19.8  (9.9, 39.6) | | Ref | 12.4  (6.4, 23.8) | Ref | | | 26.1  (14.0, 48.4) | Ref | | 8.0  (3.6, 17.8) | Ref | | | 8.4  (3.8, 18.7) | | Ref |  |  |
| **Combined work/student** |  | |  |  |  | | |  |  | |  |  | | |  | |  |  |  |
| Part/full-time student or part/full-time job | 30.2  (17.9, 51.1) | | 1.0  (0.4, 2.3) | 12.9  (6.9, 23.9) | 0.8  (0.3, 1.8) | | | 23.5  (13.0, 42.4) | 2.4  (0.7, 8.6) | | 8.7  (4.2, 18.2) | 1.2  (0.4, 3.7) | | | 7.9  (3.5, 17.5) | | 2.5  (0.5, 12.4) |  |  |
| Not a student and no job | 30.1  (15.7, 57.9) | | Ref | 17.1  (9.5, 30.8) | Ref | | | 9.8  (3.2, 30.3) | Ref | | 7.3  (3.1, 17.6) | Ref | | | 3.1  (0.8, 12.6) | | Ref |  |  |
| **Income** |  | |  |  |  | | |  |  | |  |  | | |  | |  |  |  |
| No income | 47.2  (28.5, 78.3) | | 2.5  (1.0, 5.8) | 16.6  (9.2, 30.0) | 1.1  (0.5, 2.7) | | | 18.1  (8.1, 40.3) | 1.1  (0.4, 3.2) | | 7.2  (3.0, 17.4) | 0.7  (0.2, 2.4) | | | 4.7  (1.5, 14.5) | | 0.8  (0.2, 3.6) |  |  |
| Any Income | 19.1  (9.5, 38.2) | | Ref | 14.5  (7.8, 27.0) | Ref | | | 16.6  (7.9, 34.8) | Ref | | 9.7  (4.6, 20.3) | Ref | | | 5.8  (2.2, 15.4) | | Ref |  |  |
| **Baseline HIV Status** |  | |  |  |  | | |  |  | |  |  | | |  | |  |  |  |
| Negative | 29.8  (19.0, 46.7) | | Ref | 13.2  (8.1, 21.5) | Ref | | | 21.6  (12.8, 36.5) | Ref | | 7.9  (4.3, 14.7) | Ref | | | 5.7  (2.7, 12.0) | | Ref |  |  |
| Positive | 29.0  (10.9, 77.4) | | 1.0  (0.3, 2.9) | 22.2  (9.2, 53.3) | 1.7  (0.6, 4.6) | | | 7.4  (1.0, 52.3) | 0.3  (0.0, 2.6) | | 8.2  (2.1, 32.8) | 1.0  (0.2, 4.7) | | | 10.4  (2.6, 41.8) | | 1.8  (0.4, 8.8) |  |  |
| **Initiated PrEP During Follow-up** | |  | |  |  | | |  |  | |  |  | | |  | |  |  |  |
| No | 31.4  (16.3, 60.4) | | Ref | 10.7  (4.8, 23.9) | Ref | | | 13.5  (5.1, 36.0) | Ref | | 10.5  (4.7, 23.3) | Ref | | | 7.1  (2.7, 19.0) | | Ref |  |  |
| Yes | 28.5  (15.3, 53.0) | | 0.9  (0.4, 2.2) | 15.2  (8.2, 28.3) | 1.4  (0.5, 3.9) | | | 28.4  (15.3, 52.8) | 2.1  (0.7, 6.7) | | 5.8  (2.2, 15.5) | 0.6  (0.2, 2.0) | | | 4.5  (1.5, 14.1) | | 0.6  (0.1, 2.8) |  |  |
| **Receptive condomless anal intercourse, past 3 months** | | | |  |  | | |  |  | |  |  | | |  | |  |  |  |
| No | 21.8  (12.1, 39.4) | | Ref | 14.1  (8.2, 24.3) | Ref | | | 9.8  (4.1, 23.5) | Ref | | 8.4  (4.2, 16.8) | Ref | | | 7.8  (3.7, 16.4) | | Ref |  |  |
| Yes | 61.4  (34.0, 110.9) | | 2.8  (1.2, 6.5) | 11.3  (4.3, 30.2) | 0.8  (0.3, 2.5) | | | 43.4  (21.7, 86.7) | 4.4  (1.5, 13.6) | | 2.7  (0.4, 19.4) | 0.3  (0.0, 2.6) | | | 6.0  (1.5, 24.0) | | 0.8  (0.2, 3.7) |  |  |
| **Number of male partners in past 3 months** | |  | |  |  | | |  |  | |  |  | | |  | |  |  |  |
| 0-2 | 27.7  (16.7, 45.9) | | Ref | 15.3  (9.4, 25.0) | Ref | | | 19.8  (11.0, 35.7) | Ref | | 8.2  (4.3, 15.7) | Ref | | | 3.8  (1.4, 10.0) | | Ref |  |  |
| 3+ | 44.6  (20.0, 99.2) | | 1.6  (0.6, 4.1) | 14.5  (4.7, 44.8) | 0.9  (0.3, 3.2) | | | 23.6  (7.6, 73.2) | 1.2  (0.3, 4.3) | | 14.3  (4.6, 44.3) | 1.7  (0.5, 6.5) | | | 28.7  (11.9, 68.9) | | 7.6  (2.0, 28.4) |  |  |
| **Any female partners, past 12 months** | |  | |  |  | | |  |  | |  |  | | |  | |  |  |  |
| No | 36.2  (23.9, 55.0) | | Ref | 14.7  (9.0, 24.0) | Ref | | | 22.7  (13.4, 38.3) | Ref | | 9.7  (5.4, 17.5) | Ref | | | 6.5  (3.1, 13.6) | | Ref |  |  |
| Yes | 6.0  (0.8, 42.4) | | 0.2  (0.0, 1.2) | 14.1  (5.9, 33.9) | 1.0  (0.4, 2.6) | | | 6.0  (0.9, 42.6) | 0.3  (0.0, 2.0) | | 2.7  (0.4, 19.2) | 0.3  (0.0, 2.2) | | | 6.0  (1.5, 23.9) | | 0.9  (0.2, 4.4) |  |  |
| **Transactional sex, past 12 months** | |  | |  |  | | |  |  | |  |  | | |  | |  |  |  |
| No | 35.2  (22.7, 54.5) | | Ref | 12.7  (7.5, 21.4) | Ref | | | 24.5  (14.5, 41.3) | Ref | | 5.2  (2.3, 11.6) | Ref | | | 5.6  (2.5, 12.4) | | Ref |  |  |
| Yes | 13.0  (3.3, 52.0) | | 0.4  (0.1, 1.6) | 29.2  (13.9, 61.2) | 2.3  (0.9, 5.7) | | | 0.0  (--) | -- | | 23.7  (10.7, 52.8) | 4.6  (1.5, 14.1) | | | 12.0  (3.9, 37.1) | | 2.1  (0.5, 8.6) |  |  |
| **Injection drug use, past 6 months** | |  | |  |  | | |  |  | |  |  | | |  | |  |  |  |
| No | 4.8  (0.7, 34.1) | | Ref | 19.2  (9.6, 38.5) | Ref | | | 10.5  (2.6, 42.0) | Ref | | 11.6  (4.8, 27.8) | Ref | | | 6.9  (2.2, 21.3) | | Ref |  |  |
| Yes | 29.6  (4.2, 210.0) | | 6.2  (0.4, 98.4) | 22.9  (3.2, 162.5) | 1.2  (0.1, 9.5) | | | 0.0  (--) | -- | | 0.0  (--) | -- | | | 25.5  (3.6, 181.3) | | 3.7  (0.4, 35.8) |  |  |
| **Any drug use, past 6 months** |  | |  |  |  | | |  |  | |  |  | | |  | |  |  |  |
| No | 39.4  (25.7, 60.4) | | Ref | 12.2  (6.9, 21.5) | Ref | | | 23.3  (13.5, 40.1) | Ref | | 6.8  (3.3, 14.3) | Ref | | | 5.3  (2.2, 12.0) | | Ref |  |  |
| Yes | 8.3  (2.1, 33.1) | | 0.2  (0.0, 0.9) | 19.6  (10.2, 37.6) | 1.6  (0.7, 3.8) | | | 8.9  (2.2, 35.4) | 0.4  (0.1, 1.7) | | 10.5  (4.4, 25.2) | 1.5  (0.5, 4.8) | | | 8.4  (3.2, 22.4) | | 1.6  (0.4, 5.9) |  |  |
| **Binge drinking (5+ drinks) on 5 or more days, past 30 days** | | | |  |  | | |  |  | |  |  | | |  | |  |  |  |
| No | 28.0  (17.1, 45.6) | | Ref | 16.2  (10.2, 25.7) | Ref | | | 20.8  (11.8, 36.7) | Ref | | 8.5  (4.6, 15.8) | Ref | | | 7.3  (3.7, 14.7) | | Ref |  |  |
| Yes | 31.8  (13.2, 76.3) | | 1.1  (0.4, 3.1) | 7.7  (1.9, 31.0) | 0.5  (0.1, 2.1) | | | 12.4  (3.1, 49.5) | 0.6  (0.1, 2.7) | | 3.8  (0.5, 27.3) | 0.5  (0.1, 3.5) | | | 4.0  (0.6, 28.4) | | 0.5  (0.1, 4.4) |  |  |

Caption: PrEP = pre-exposure prophylaxis;
